# FluxTransgenics: a flexible LIMS-based tool for management of plant transformation experimental data

**DOI:** 10.1186/1746-4811-10-20

**Published:** 2014-06-26

**Authors:** Lucas AF Hanke, Cristiano S Botelho, Fernando AF Braz, Paulo HS Batista, Aurea V Folgueras-Flatschart, Roberto W Noda, Andrea A Carneiro, Alessandra C Faria-Campos, Sérgio VA Campos

**Affiliations:** 1 Department of Computer Science, Universidade Federal de Minas Gerais, Antônio Carlos Avenue, 6627, Pampulha, 30123-970 Belo Horizonte, MG, Brazil; 2 Embrapa Maize and Sorghum, MG 424 Highway KM 45, Sete Lagoas, MG, 35701-970, Brazil; 3 INMETRO, Nsa.Sra.das Graças Avenue, 50, 25250-020 Xérem-Duque de Caxias, RJ, Brazil

**Keywords:** Laboratory information management system, Workflow, Plant transformation, Maize, Sorghum

## Abstract

**Background:**

The production and commercial release of Genetically Modified Organisms (GMOs) are currently the focus of important discussions. In order to guarantee the quality and reliability of their trials, companies and institutions working on this subject must adopt new approaches on management, organization and recording of laboratory conditions where field studies are performed. Computational systems for management and storage of laboratory data known as Laboratory Information Management Systems (LIMS) are essential tools to achieve this.

**Results:**

In this work, we have used the SIGLa system – a workflow based LIMS as a framework to develop the FluxTransgenics system for a GMOs laboratory of Empresa Brasileira de Pesquisa Agropecuária (EMBRAPA) Maize and Sorghum (Sete Lagoas, MG - Brazil). A workflow representing all stages of the transgenic maize plants generation has been developed and uploaded in FluxTransgenics. This workflow models the activities involved in maize and sorghum transformation using the *Agrobacterium tumefaciens* method. By uploading this workflow in the SIGLa system we have created *Fluxtransgenics*, a complete LIMS for managing plant transformation data.

**Conclusions:**

FluxTransgenics presents a solution for the management of the data produced by a laboratory of genetically modified plants that is efficient and supports different kinds of information. Its adoption will contribute to guarantee the quality of activities and products in the process of transgenic production and enforce the use of Good Laboratory Practices (GLP).

The adoption of the transformation protocol associated to the use of FluxTransgenics has made it possible to increase productivity by at least 300%, increasing the efficiency of the experiments from between 0.5 and 1 percent to about 3%. This has been achieved by an increase in the number of experiments performed and a more accurate choice of parameters, all of which have been made possible because it became easier to identify which were the most promising next steps of the experiments. The FluxTransgenics system is available for use by other laboratories, and the workflows that have been developed can be adapted to other contexts.

## Background

The amount of data generated by experimental processes has been growing significantly. As a consequence, the management of data and processes from laboratories has been the subject of debate. Stafford [1] has discussed the need of computational tools designed to manage all laboratory information — including, but not limited to, the data produced by these laboratories — highlighting the relevance of such systems. These applications are referred to as Laboratory Information Management Systems (LIMS) and are characterized by data storage and tracking functionalities, management of the laboratory processes, quality assurance and integration with other systems and equipments. Several LIMS are currently available as academic, proprietary and open source solutions. Some examples of these include SQL LIMS [2], LabSoft [3], LabWare [4], FreeLIMS [5] and the systems developed by Hendrick [6], Quo [7], Tharayil [8] and Sanchez [9].

The available LIMS are usually restricted to the needs of the laboratories involved in their development and are specific to the context of these laboratories. SQL LIMS, for example, has distinct solutions for pharmaceutical, chemical, nourishing, forensic and water analysis laboratories. Other systems present functionalities varying from data management from an academic micro chip fabrication facility [6] or a cancer research laboratory [7], to a maize mapping project [9] or a service based LIMS focused on the integration of the data stored in biological databases [8]. These systems have been designed to manage specific data for one kind of laboratory and therefore present some limitations when customization is necessary.

Although a significant number of LIMS is available, its use is still limited since most of them are designed especially to the needs of the supported laboratory. Laboratories of transgenic production and analysis are among the laboratories for which only a small number of LIMS have been developed. One example of such system is Villager (Villager Transgenic Animal Management System), a proprietary solution for the management of information related to the development of genetically modified animals [10]. Currently there are only a few similar systems for laboratories working on transgenic plants, although most of them can not be characterized as LIMS. These systems include Phytotracker [11] and QTreds [12], which we compare with the SIGLa system in Table 1. Flexible LIMS can be used to manage these kind of data. Melo [13] has proposed the **SIGLa** system, a flexible workflow based LIMS designed to allow its adaptation to activities and processes of different types of laboratories. A workflow can be defined as the tasks executed according to a set of rules and procedures in order to conclude a process. A workflow consists of a sequence of activities, or a complex set of processes occurring concurrently and eventually impacting in others [14]. Other LIMS incorporate this concept, such as the system developed by Wilkins [15], which manage data from proteomics laboratories.

**Table 1:** **Table containing a comparison between *FluxTransgenics * and similar systems**

**Feature**	***FluxTransgenics***	***Phytotracker***	***QTreds***
Operational system	Web Application, can be accessed from any OS.	Mac OS or Microsoft Windows.	Web Application, can be accessed from any OS.
Data storage	MySQL Relational Database.	FileMaker Pro.	MySQL Relational Database.
Process support	Most laboratory processes. Adaptable to any workflow.	Pre-defined workflow.	Adaptable to any workflow.
Access control	Permissions are granted to user or roles. Roles, or groups, can be created by administrator user.	Permissions are granted only to users. The concept of roles is non existent.	Permissions are granted to user or roles. But the roles are fixed (cannot be created).

Furthermore, the SIGLa system shares some key features with Electronic Laboratory Notebooks (ELN), such as record keeping, online data sharing and retrieval, support to different types of data formats, collaboration, permissions system, inventory and file management and audit trail. The SIGLa system is web based but can also operate offline by simply installing the complete client-server architecture in a stand alone machine. There are other ELNs available, such as eJournal [16,17], eCAT [18] and E-WorkBook Suite (EWBS) [19], and even the web application Evernote [20] that can be used to manage data. However, these systems lack the flexibility and workflow perspective of LIMS-based systems such as SIGLa.

In this work, we propose the construction of workflows to be used in the SIGLa system for data management of a plant transformation facility and modifications in SIGLa to comply with the needs of these complex processes. By uploading the workflows to the SIGLa system the LIMS system is created. These workflows can serve as a model for similar laboratories. The system proposed is called *FluxTransgenics* and has been designed to manage data of the Cellular Biology and Plant Tissue Culture Laboratory at EMBRAPA Maize and Sorghum — MG — Brazil. This facility works on the analysis and production of transgenic maize and sorghum plants. A complex workflow has been constructed for data management considering one of the main methods used for maize transformation: the use of the bacteria *Agrobacterium tumefaciens*. Transformation through *A. tumefaciens* uses the bacteria natural system of gene transferring [21]. This process has a significant number of activities with specific attributes that are defined with great detail during workflow construction.

*FluxTransgenics* has been developed in collaboration with EMBRAPA Maize and Sorghum — MG — Brazil, an institution that works, among other things, in the production of transgenic plants. EMBRAPA has initiated the use of the Good Laboratory Practices (GLP) to fulfill the demand for quality at the production and commercialization of genetically modified organisms. GLP are a set of management principles for research laboratories activities that provide a framework within which studies are planned and performed in order to ensure results consistency and reliability [22]. In order to enforce the GLP adoption it is important to demonstrate that information produced and processes applied to obtain it are correct and traceable. The use of automated systems as **FluxTransgenics** to follow each step of the research in a laboratory is essential to ensure the quality of the production.

The *FluxTransgenics* system has been used to manage data from experiments starting on September 2011. Up to September 2012, it has stored data of 67 experiments, including complete and ongoing experiments. From these experiments, 20 transgenic plants have been successfully generated. These 67 experiments have been performed during the initial setup phase of the transformation laboratory. The small number of plants generated is a consequence of the problems encountered during the protocol standardization. Currently the laboratory produces between 6 to 15 plants per experiment, which generates an amount of data that requires more efficient management tools.

Our next step in the research is to perform a data mining analysis of this data, searching for patterns that could identify the ideal conditions for a successful experiment. It is important to note that this analysis will be possible only because the data is stored in a structured representation, where not only each experiment is stored, but also which experiment is derived from which one, making it possible to track and relate all experimental data.

Access to the *FluxTransgenics* system is available at the address http://www.luar.dcc.ufmg.br/FluxTransgenics(login/password: guest/guest). The definition of the transformation process is completely included in the workflow, modifications for other protocols require only a change in the workflow definition. So, adaptations for other laboratories do not require changes in the source code of the system, only in its description. The workflow definition is available for download at the address above. In addition to this, the source code of the SIGLa system is also available for code developers as well.

## Methods

### ***The SIGLa system***

**Figure 1:**
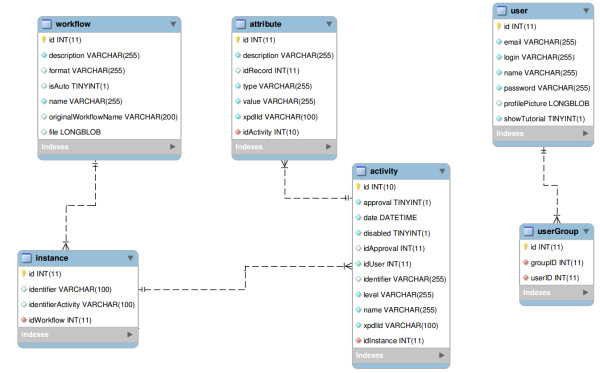
**ER Diagram.** Simplified Entity-relationship Diagram with the main entities fo the system.

SIGLa has been constructed using *Java* technology and uses MySQL as a database server and Apache Tomcat as a Web server. The system has a web interface which is platform-independent and can be accessed using the main browsers (Internet Explorer, Google Chrome, Mozilla Firefox and Safari). Workflow files are uploaded in the system through its web interface. The activities created in the workflow construction are interpreted by the system as links. The workflow of **FluxTransgenics** has been constructed using the Together Workflow Editor (TWE) [23] in the XML Process Definition Language (XPDL) file format. The SIGLa system can represent different types of workflows which have been modeled in TWE. In this application, the protocols are defined as processes that are composed of steps, referred to as activities. An activity represents events of a process and, as such, has transitions, actors and rules. This information is represented as attributes in the workflow definition. Therefore, the user can define the characteristics of the attributes of each activity, such as its types, the range of values that each attribute can assume, its formats or even define auto-calculated attributes derived from other attributes. In TWE, inputs and outputs of each activity are also defined, including the relation of these with the experiments. During workflow definition it is also possible to assign to each activity a documentation that contains standard protocols, instruments calibration, procedures and records associated with the activities.All this information is stored in a entity-relationship database. However, that it is not a traditional entity-relationship diagram, because the SIGLa system is completely dynamic, and a hard-coded ER model would not allow different activities with different attributes to be stored without modifications to the source code. Instead, an activities table store all the data, using metadata to indicate, whenever necessary, to which experiment or activity each data belongs to. A simplified entity-relationship diagram is show in Figure 1.

### ***The construction of the FluxTransgenics LIMS***

The construction of the FluxTransgenics LIMS consisted of the development of the plant transformation workflow and its upload to the SIGLa system. In this workflow each activity corresponds to an experimental step, and its attributes identify the types of information that are generated in this experimental step. The sequence of activities then contain all the steps of the transformation protocol. The attributes in each activity store the specific information of that activity. As a consequence, the workflow has the complete description of the data being managed, all the steps and for each step the required data.

Because of this, the FluxTransgenics system has been fully developed by specifying the workflow, without changing the code of the SIGLa system. Other workflows, or adaptations to this workflow can be developed by changing the workflow files in the editor, without changing the SIGLa system. The information managed by the FluxTransgenics system is completely contained in its workflow, the SIGLa system is the engine that drives the workflow, in the same way as experimental data can be stored in an Excel spreadsheet file. This file contains the data and all of the formulas that are required to understand it, the Excel program is merely an engine to understand this data.

There are several different types of attributes, each fulfilling a specific need in storing and managing laboratory data. For example, to specify which reagents are used in an experimental step, one attribute “reagent” can be specified. This information is entered by the user when registering the experiment. There are several ways in which a reagent can be specified. One way is to let the user type a string with the reagent name. Another is to let the name be chosen from a list of specified names. Yet another is to let the user determine which are the valid reagent names in another menu. Which option will be used is specified in the workflow.

Other features of the SIGLa system can be defined in the workflow, such as the protocol used, or the callus identifiers. Protocols are usually specified in a type of attribute, called *register*, which lets the user choose from a list of pre-specified names. Each name, when defined, can allow a text file (in plain text, Word or any other format) to be stored with the description of the protocol. In this way, a list of allowed protocols is stored, together with the full information about the protocol.

Callus identifiers use a different feature, called *auto increment*. When an attribute has this type, its value is not entered by the user, but defined when the activity is registered, as a unique identifier generated by the SIGLa engine. In this way the system guarantees its uniqueness, and can even generate bar codes to be attached to the samples, if needed.

This information is stored by the SIGLa system in dynamically defined tables in the database. The entities in this database are activities, which are related to one another by the relation *is derived from*, with a cardinality of 1:n, i.e., an activity can generate several “daughter” activities, but each activity has exactly one “parent” activity, except for the first activity. For example, the second activity in an experiment *is derived* from the first activity, but this first activity is not derived from any other.

Tables have a dynamic content, because each activity is defined by different attributes. So each item stored in the attributes table is a line containing, basically, the tuple (*activity*,*attribute*_*name*,*value*), where *activity* defines which activity in the workflow it refers to, *attribute_name* defines which attribute, and *value* defines the value of the attribute entered by the user. The SIGLa system is responsible for reconstructing, from the activities table and an experiment identifier, the data pertaining to that experiment.

It is important to emphasize that the SIGLa system is used in this work as an *engine* that drives the plant transformation workflow creating the FluxTransgenics LIMS. All of the information relating to plant transformation and experimental data is stored in the workflow.

### Workflow construction and transformation protocol

**Figure 2:**
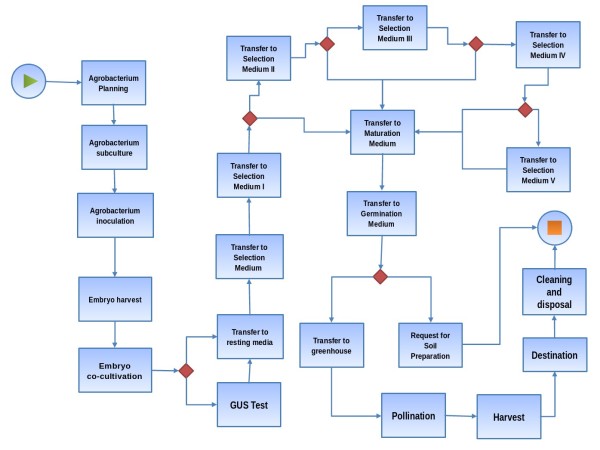
***Agrobacterium tumefaciens’s * Transformation *Workflow *. ***Workflow* of the maize and sorghum transformation process by *Agrobacterium tumefaciens*. Each box represents an activity in the process and the arrow from an activity to the other represents a transition.

The workflow has been constructed considering all steps of a transformation method, as shown in Figure 2. The protocol used for *Agrobacterium*-mediated transformation of immature zygotic embryos is based on the work of Frame *et al.*[24] and Vega *et al.*[25]. For details on the protocol used, please see the supplementary material available at the address http://www.luar.dcc.ufmg.br/FluxTransgenics.

**Figure 3:**
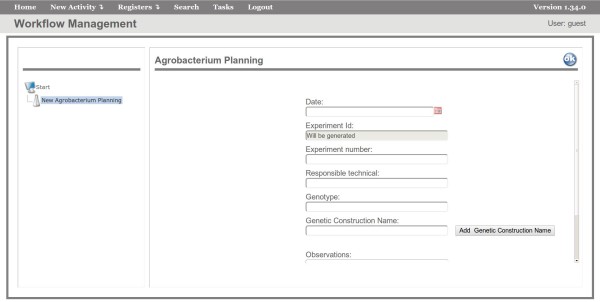
**Planning Activity.** Interface of the FluxTransgenics system showing the execution of the first activity of the workflow — *Planning*.

*FluxTransgenics* contains a workflow constructed to represent all the steps of the transformation process performed at the Cellular Biology and Plant Tissue Culture Laboratory at Embrapa Maize and Sorghum using *A. tumefaciens*. This workflow allows recording and managing reagents and protocols used in the transformation process, including the clones and modified organisms produced. The initial activity in the workflow is *Planning* (Figure 3), which is used to record general information on the plant transformation experiment, such as genotype, experiment number, username, genetic construction name, etc. In this activity, an experiment identifier (id) is assigned to each experiment (using the *auto increment* feature), which is used to link all data related to the experiment and allow experiment tracking. In the transformation using *A. tumefaciens* (TAT), the activity *Planning* includes the input of information regarding the Genetic Construction that is selected from a list of constructions names previously stored in a table of the system’s database (using the *register* feature).

**Figure 4:**
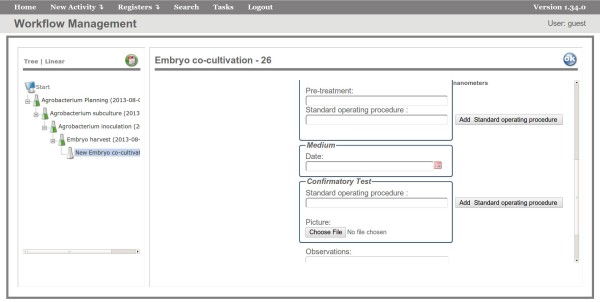
**Embryo Co-cultivation Activity.** Interface of the FluxTransgenics system showing the Embryo co-cultivation activity. After it has been executed, information about absorbance, amount and size of embryos, confirmatory test, protocols, etc is stored. In the user interface, activities that have already been executed are represented as green (filled) tubes, while activities that are available to be executed are shown as white (empty) tubes.

The next activities are *Agrobacterium subculture* and *Agrobacterium inoculation* in which the system stores the procedure date and the protocols used. In *Embryo harvest*, the main information is the collection date. In *Embryo Co-cultivation*, the collected embryos from the previous activity are genetically modified and all information on those is stored, such as length and amount of embryos, culture medium and confirmatory test (Figure 4). After the execution of the *Embryo Co-cultivation* activity, there are two different paths. In some cases, a *GUS Test* is performed to check the quality of the infectious process and, only after this test, the next activity is executed, while in other cases, the *GUS Test* is not performed and the next activity is *Transfer to the Resting Medium*. In the maize and sorghum transformation experiments, the embryos remain for some time in a culture medium and can be transferred from up to five different media to perform the selection. The transfer to each medium constitutes a new activity in the workflow with its attributes.

**Figure 5:**
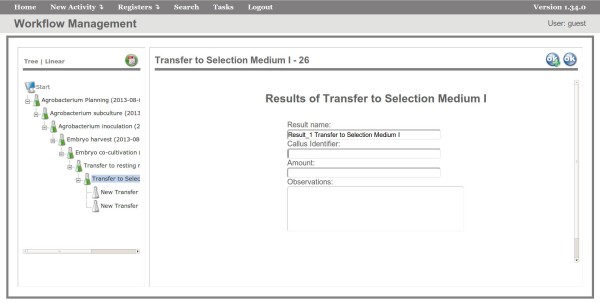
**Interface for registering calluses.** Interface of the FluxTransgenics system showing the forms for filling the outputs of the Transfer to Selection Medium I.

**Figure 6:**
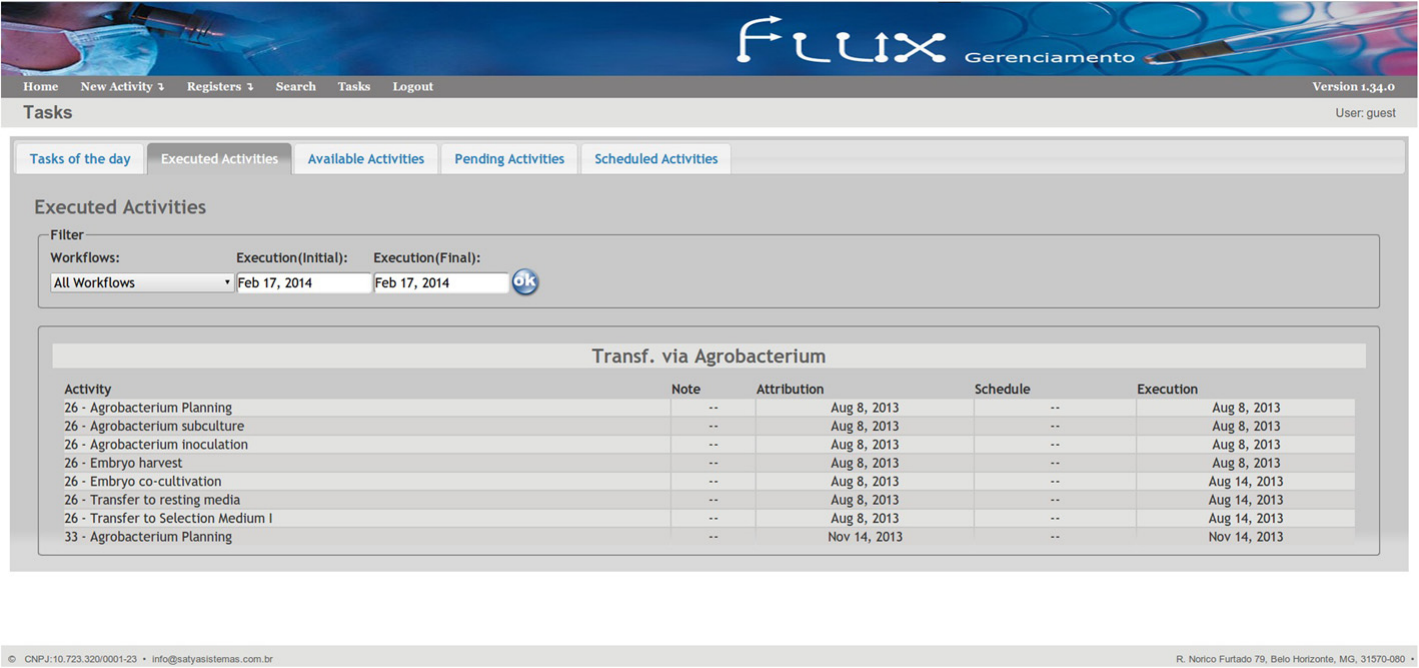
**Tasks’s Screen.** Interface of the Tasks’ screen showing the executed activities.

In each transfer to a different medium, a control of the generated calluses is performed through the recording of inputs and outputs. This information contains the callus’ *ID* and the number of the copies of each callus (Figure 5). In the *Transfer to A Medium* activity, the stored calluses are identified and used (all or part of it) as input to the next activity. This behavior is repeated until the activity *Transfer to Greenhouse*. The following activity is the *Pollination*, where the pollination type used (self, cross or sibling) can be chosen from a drop-down menu. *Pollination* outputs are the id of the maize ear and the number of seeds. After the *Pollination*, the next activity is *Harvesting*. In this activity, the amount of picked seeds is stored as an output, including the date and the protocol used. The final activity is *Destination*, which stores the date and the protocol of the execution, allowing the user to record the destination of the seeds. This *input/output* feature is an important issue in FluxTransgenics. Through this procedure, the user can keep track of which calluses will generate the final genetically modified plants.During the laboratory routine, the user can access the system and verify, through the the tasks’ screen, which activities have been executed, which are available e which are scheduled to be executed (Figure 6).

The FluxTransgenics workflow — developed to represent the transformation process — presents a comprehensive set of attributes designed to allow recording and managing of all activities performed during the transformation process and the definition of the details regarding those activities for an easy retrieval by the user for reports production.

## Discussion

The *FluxTransgenics* system has been successfully tested by the researchers at EMBRAPA Maize and Sorghum as a tool to track the plant transformation process.

The process of producing GMO plants is complex involving a large number of steps and it may take a long time. For maize, for example, it may take up to six months to complete an experiment. During this time, researchers are looking for what is called an elite event that is, a result that has the ideal characteristics to produce the desired outcome. An elite event must contain a small number of copies of the implanted gene, express the heterologous protein at high levels, and this protein must be the only difference between this new lineage and the previous one. In our lab, about twenty events are produced for each gene, in search of an elite event.

However, due to the long duration of the experiments, it is not always possible to wait until it is known that an event will be a successful one before initiating a new one and therefore several experiments are performed simultaneously. An accurate management of the samples and experimental steps is necessary to be able to identify the more promising results. However, with so many different ongoing experiments being performed by different laboratory analysts, the link between an elite event and its related experiments can be easily lost. The use of standard protocols and computational aids are essential to follow results efficiently and accurately.

By using the protocol developed by [24-26], and implementing it using FluxTransgenics, EMBRAPA’S lab has been able to increase the number of experiments performed at it from one to two a week to up to 16 (4 transformations with 4 different genic constructions) after protocol standardization. It has become much more efficient to identify the best conditions to generate a transgenic event and to coordinate the different steps of the various experiments to be performed daily. Moreover, modifications of the protocols can not only be easily implemented, but also can be easily related to which experiments use it instead of the original ones.

Previously, the efficiency of the transformation process at EMBRAPA’S lab was very low, only between 0.5 and 1 percent of the experiments were successful. As a consequence of the work described, the efficiency has increased to about 3 percent, an increase of 300 to 600 percent.

Sixty-seven experiments have been performed and twenty transgenic plants have been produced using *FluxTransgenics* to manage the steps of the process. These experiments represent one year of experimental data that have been successfully stored in the system.

In spite of the low productivity of the initial experiments, the use of an automated management system was very important. Because so few experiments were successful, identifying those and the reasons for their success was crucial for the establishment of a productive laboratory. Current productivity in the laboratory has since increased to performing on average one experiment a day, and each experiment generating from 6 to 15 plants.

A new version of the system is under development with additional features, including a workflow for management of data from transformation performed using microparticles bombardment and the design of new workflows to manage processes that are indirectly related to the processes of plant transformation.

*FluxTransgenics* is a LIMS that has been customized to attend the needs of the Cellular Biology and Plant Tissue Culture Laboratory at EMBRAPA Maize and Sorghum. It is currently being used in its production. It can be accessed at the URL: http://www.luar.dcc.ufmg.br/FluxTransgenics (login/password: guest/guest). At this address the system can be used to store a limited amount of experimental data, and to access the system source code.

The system can be easily adapted to similar laboratories by modifying the workflow description. A description of how to adapt the workflow to other laboratories is outside the scope of this paper, but is available at the address given. Potential users are welcome to use the system and contact the authors for modifications for other processes and different laboratories.

## Conclusion

The production and commercial release of Genetically Modified Organisms (GMOs) are currently the focus of important discussions. Thus, efficient management methods to assess the conditions under which GMO laboratories experiments are carried out are needed. As a developer of GMOs, EMBRAPA Maize and Sorghum (Brazil) has enforced the use of the Good Laboratory Practices (GLP) to ensure quality on the processes of producing modified genetically plants and needs a computational system to support the use of GLP.

In this work, we present *FluxTransgenics*, a system for management of data on the process of plant transformation by *A. tumefaciens* to be used as a computational aid. The system is able to manage the information and the correct sequence of activities of the transformation performed at the Cellular Biology and Plant Tissue Culture Laboratory at EMBRAPA Maize and Sorghum. It is possible by using *FluxTransgenics* to track the stored data during the execution of each activity of the plant transformation, ensuring the quality required by GLP.

## Competing interests

The authors declare that they have no competing interests.

## Authors’ contributions

RWN and AAC and AVFF were involved in modeling, validation of the workflows and tests of the LIMS. LAFH, ACFC and CSB were directly involved in workflow construction, contributed in SIGLa System alterations and participated of all other previously cited work phases. PHSB, FAFB and CSB have modified SIGLa code to develop new functions for the system, according to the transgenic laboratory needs. SVAC is the principal investigator and supervised all the work as well as manuscript preparation. All authors read and approved the final manuscript.
